# Cardiac sympathetic afferent ablation to prevent ventricular arrhythmia complicating acute myocardial infarction by inhibiting activated astrocytes

**DOI:** 10.1111/jcmm.17508

**Published:** 2022-08-07

**Authors:** Jugang Chen, Yingjie Chu, Meng Gao, Xin Dai, Bin Li, Xiufen Qu, Dechun Yin

**Affiliations:** ^1^ Department of Cardiology The First Affiliated Hospital of Harbin Medical University Harbin China; ^2^ Department of Cardiology Henan Provincial People's Hospital, People's Hospital of Zhengzhou University Zhengzhou China; ^3^ Department of Oncology The First Affiliated Hospital of Harbin Medical University Harbin China

**Keywords:** acute myocardial infarction, astrocyte, cardiac sympathetic afferent nerves, paraventricular nucleus, ventricular arrhythmias

## Abstract

Enhanced cardiac sympathetic afferent reflex (CSAR) contributes to ventricular arrhythmia (VA) after acute myocardial infarction (AMI). However, central regulation mechanisms remain unknown. The aim of this study was to investigate whether local cardiac sympathetic afferent ablation (LCSAA) could reduce VA by inhibiting activated astrocytes in the hypothalamus paraventricular (PVN) in an AMI rat model. The rats were randomly divided into AMI, AMI + BD (baroreceptor denervation), AMI + LCSAA and AMI + BD+ LCSAA groups. Before the generation of AMI, BD and (or) LCSAA were performed. At 24 h after AMI, the incidence and duration of VA in AMI + LCSAA group and AMI + BD + LCSAA group were significantly reduced than AMI group (*P < 0.05*). Furthermore, LCSAA significantly reduced GFAP (a marker for activated astrocytes) positive cells and their projections as well as the level of TNF‐α and IL‐6 in the PVN of AMI + LCSAA group and AMI + BD+ LCSAA group, along with the decrease of neuronal activation in PVN and sympathetic nerve activity (*P < 0.05*). but BD had no obvious difference between AMI + LCSAA and AMI + BD + LCSAA group (*P > 0.05*). Therefore, LCSAA could decrease sympathoexcitation and VA occurrence in AMI rats by inhibiting astrocyte and neuronal activation in the PVN. Our study demonstrates that activated astrocytes may play an important role on CSAR in AMI.

## 1 INTRODUCTION

Malignant ventricular arrhythmias (VA) complicating acute myocardial infarction (AMI) is the most common cause of sudden cardiac death (SCD).^1^ Sympathetic overactivation contributes to initiation of VA complicating AMI.^2^ Inhibit sympathetic activation has become an important prevention and treatment strategy for VA and SCD.^3^ Cardiac sympathetic afferent reflex (CSAR) may play an important role on this context; however, the central regulation mechanisms are incompletely understood.

Cardiac sympathetic afferent reflex (CSAR) is a sympathoexcitatory reflex with positive feedback characteristics. The precise neural conduction pathways of CSAR have been not well understood, but the hypothalamus paraventricular (PVN) is regarded as an important integrative central site of CSAR.^4^ Some research has reported that cardiac sympathetic afferent nerves (CSAN) activated by myocardial ischemia signal increase the inflammatory cytokines in PVN.^5^ Our previous study has suggested that activated astrocytes in PVN increased the inflammatory cytokines production at the early phase of AMI.^6^ Therefore, it is conceivable that astrocytes activation in PVN may involve in CSAN response to AMI.

Glial fibrillary acidic protein (GFAP) is an intermediate filament protein peculiar to astrocyte and upregulation of GFAP indicated the activated form of astrocyte.^7^ Many studies have shown the close interactions between neuronal activity and astrocytic plasticity provide a dynamic astrocyte–neuron network, which then regulates GFAP expression at multiple layers under disease conditions.^8^, ^9^ Based on these considerations, we hypothesized that the excitation of CSAN would result in neuronal and inflammation‐associated GFAP cells activation in PVN after AMI, then promoting sympathetic overactivation and the occurrence of VA complicating AMI.

## 2 MATERIALS AND METHODS

### 2.1 Animals

Male Sprague–Dawley rats (270‐306 g) were purchased from the experimental animal centre at the Second Affiliated Hospital of Harbin Medical University, China. All experimental protocols were approved by the Institutional Animal Care and Use Committee of Harbin Medical University. All animals were acclimated to new situation for seven days before experiment. They were raised by two animals per a cage with 12 h light and free access to food and water in a climate‐controlled room.

Eighty rats were anesthetized by intraperitoneal injection of a mixture of α‐chloralose (40 mg kg^−1^) and urethane (800 mg kg^−1^), then each animal was intubated and mechanically ventilated using a rodent ventilator (683, Harvard, Millis, MA, USA) with room air (75–80 breaths/min). The core body temperature was maintained at 37 ± 0.3 °C with heating pads.

### 2.2 Generation of AMI model and nerve ablation

Eighty rats were randomly divided into the groups of AMI (*n* = 20), AMI + BD (baroreceptor denervation, *n* = 20), AMI + LCSAA (local cardiac sympathetic afferent ablation, *n* = 20) and AMI + BD+ LCSAA groups (*n* = 20). AMI was induced by ligating the left anterior descending coronary artery as previously described.^6^ AMI was confirmed by the electrocardiograph (ECG) immediately displayed ST‐segment elevation and the lesion area gradually becoming pale. Before the generation of AMI, rats underwent one or both of the following interventions.

#### 2.2.1 Vagotomy and baroreceptor denervation

Vagotomy and baroreceptor denervation were carried out to reduce or eliminate impact on CASR, as previously reported.^10^, ^11^ Briefly, bilateral cervical vagus nerves and carotid sinus nerves were exposed, identified and sectioned. The common carotid arteries and carotid bifurcation were stripped of adventitial tissues from 4 mm below the bifurcation to 4 mm above. The remaining nerve fibres were destroyed by the 10% phenol solution. The validity of BD was evaluated by recording changes in heart rate (HR) to intravenous injection of phenylephrine (20 μg/kg), namely, mean arterial pressure increased by more than 25 mmHg while HR not exceeding 5 beat/min. Rats recovered for at least an hour after vagotomy and baroreceptor denervation or sham operation.

#### 2.2.2 Local cardiac sympathetic afferent ablation

LCSAA were performed in epicardial surface of the left ventricle with solution of 10% phenol and 70% ethyl alcohol as previously described.^5^ The phenol solution was painted to the distribution of the left anterior descending coronary artery to interrupt the cardiac sympathetic fibres innervating only that region of the heart. The other parts of the heart and surrounding tissues were protected from the spread of phenol by covering with absorbent paper and sterile gauze. The validity of LCSAA was evaluated by immunohistochemistry. AMI was induced immediately after the application of the phenol solution.

### 2.3 Heart rate variability and VA measurement

Heart rate variability (HRV) was measured via 5‐min ECG record at 24 h after coronary occlusion under anaesthesia, as described previously.^6^ Spectral power included low frequency component (LF, sympathetic components, from 0.04 to 0.15 Hz), high frequency component (HF, parasympathetic component, from 0.15 to 0.40 Hz) and ratio between LF and HF (sympathetic components, LF/HF), were analysed.

Spontaneous VA was continuously recorded for 24 h in all rats and included ventricular premature beats (VPB)、ventricular tachycardia (VT) and ventricular fibrillation (VF), as we described previously.^6^

### 2.4 Collection of blood and tissue samples

At 24 h after AMI, rats were decapitated to collect trunk blood, brain and heart tissues as described previously.^6^ Plasma and PVN tissues were separated and then stored at −80 °C for subsequent analysis. Other rats were perfused transcardially, then the samples of brain and heart tissues were collected and embedded in paraffin for histopathology study.

### 2.5 Biochemical assays

Plasma and tissue were measured using ELISA techniques, according to the manufacturer's instructions. Plasma norepinephrine (NE), PVN tissue IL‐6 and TNF‐α levels were separately measured with rat norepinephrine (NE) Kit (Cusabio Biotech Co., China), rat IL‐6 ELISA Kit (SABiosciences, USA) and rat TNF‐α ELISA Kit (Bioss Biotech Co., China).

### 2.6 Histopathology studies

Masson's trichrome stain and immunohistochemistry techniques was separately used to determine infarct size and the expression of GFAP (Glial fibrillary acidic protein, a marker for activated astrocytes), Iba1 (Ionized calcium binding adaptor molecule‐1, a marker for activated microglia) and Fra‐LI (Fra‐like, fos family gene, a marker for chronically continuous neuronal activation) in the hypothalamus PVN as well as TRPV1 (Transient Receptor Potential Vanilloid 1) in ventricular myocardium as detailed in our previous reports.^6^ Briefly, infarct size was defined as the area stained blue and calculated as the total length of the infarct area as a percentage of the total left ventricle circumference. The paraffin sections were incubated with primary antibodies (GFAP, 1:400, Bioss, China; Fra‐LI, 1:50, Santa Cruz Biotechnology; Iba1,1:2000, Abcam Ltd.; TRPV1,1:50, Abcam Ltd.) at 4 °C overnight. After washing in PBS, sections were further incubated with biotinylated secondary antibodies (IgG‐HRP, Zsbio, China). DAB substrate‐chromogen system was used for staining. For each animal, the positive cells within the bilateral borders of the PVN were analysed in three consecutive sections at about −1.80 mm from bregma.

### 2.7 Statistical analysis

Data were expressed as the mean ± standard deviation. Comparisons among continuous data was analysed by anova followed by SNK‐q test in SPSS 21.0 software (IBM Corp., USA). *P* < 0.05 was considered as statistically significant.

## 3 RESULTS

### 3.1 General results

**FIGURE 1:**
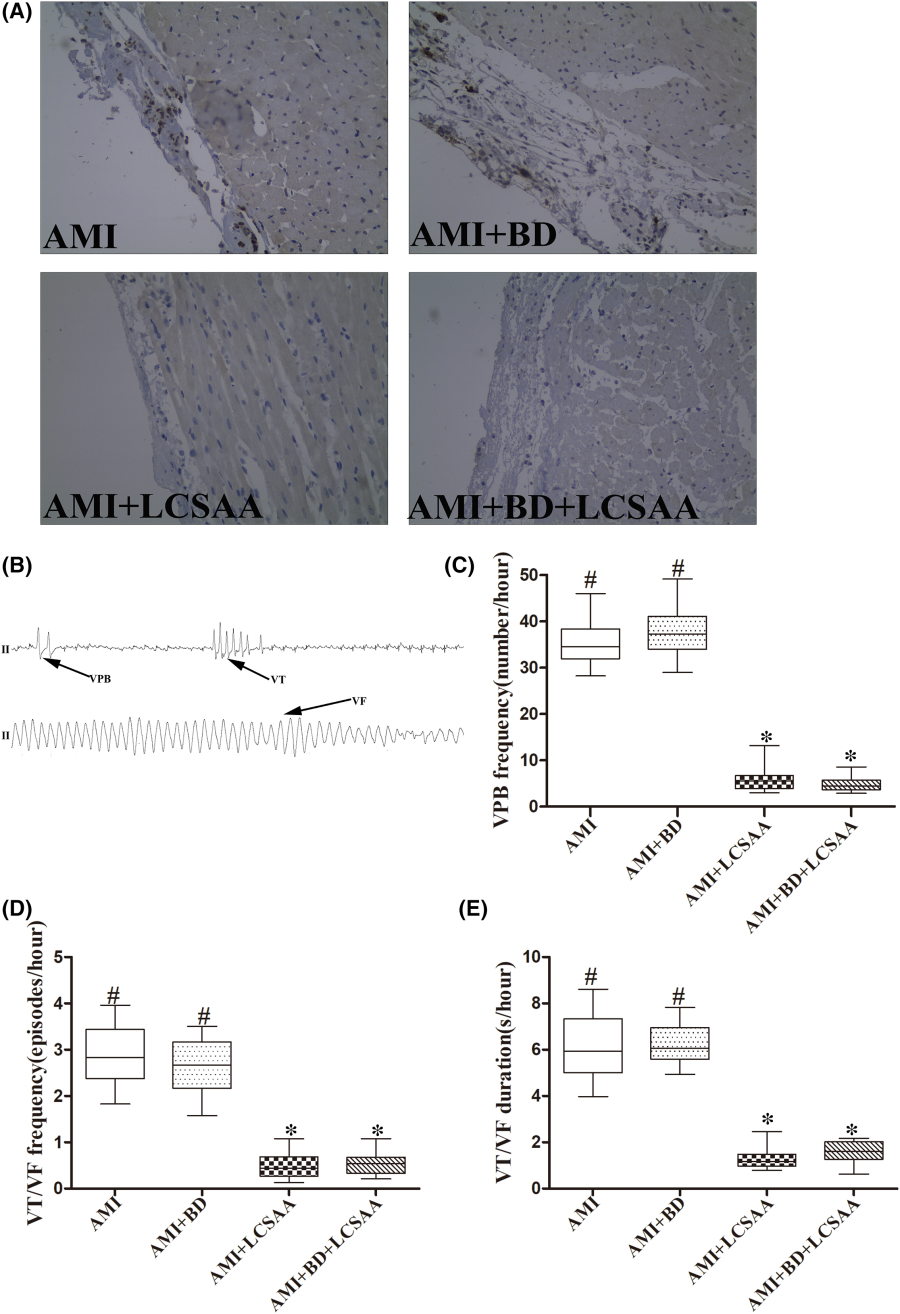
LCSAA by ablating TRPV1‐positive fibres in ventricle reduced VA occurrence. (A). TRPV1‐positive fibres in ventricle in four groups (×400, Scale bar = 40 μm). (B–E). The occurrence of the VA in the four groups at 24 h after AMI. *P < 0.05 versus AMI group; ^#^ *P* < 0.05 versus the AMI + LCSAA group. TRPV1, Transient Receptor Potential Vanilloid 1; AMI, acute myocardial infarction; LCSAA, local cardiac sympathetic afferent ablation; BD, baroreceptor denervation; VA, ventricular arrhythmia

To created nerve ablation model, three of forty rats needed a second operation for generation of vagotomy and baroreceptor denervation. CSAN ending are mainly TRPV1 positive receptors, TRPV1‐expressing afferent nerves are essential for CSAR during AMI. As shown in Figure 1A, TRPV1‐expressing afferent nerves in left ventricle of the heart of AMI + LCSAA and AMI + BD+ LCSAA groups were significantly less than in AMI group (*P* < 0.05). These observations suggest that we succeeded in creating the nerve ablation model. Unfortunately, fourteen rats died of severe bradyarrhythmia or ventricular fibrillation among generation of AMI and (or) nerve ablation model (Table 1).

**TABLE 1:** General results among the four AMI groups

Group	Death (*n*)		Research analysis (*n*)
	Bradyarrhythmia	tachyarrhythmia	
AMI (*n* = 20)	1	2	17
AMI + BD (*n* = 20)	3	2	15
AMI+ LCSAA (*n* = 20)	3	1	16
AMI + BD+ LCSAA (*n* = 20)	2	0	18

Abbreviations: AMI, acute myocardial infarction; BD, baroreceptor denervation; LCSAA, local cardiac sympathetic afferent ablation.

In AMI + LCSAA or AMI + BD+ LCSAA groups, LCSAA significantly decreased the numbers of VPB, VT/VF frequency and duration (*P* < 0.05), but BD had no obvious effects on VA(*P* > 0.05) (Figure 1B–E).

### 3.2 Activity of astrocyte and neurons in the PVN

**FIGURE 2:**
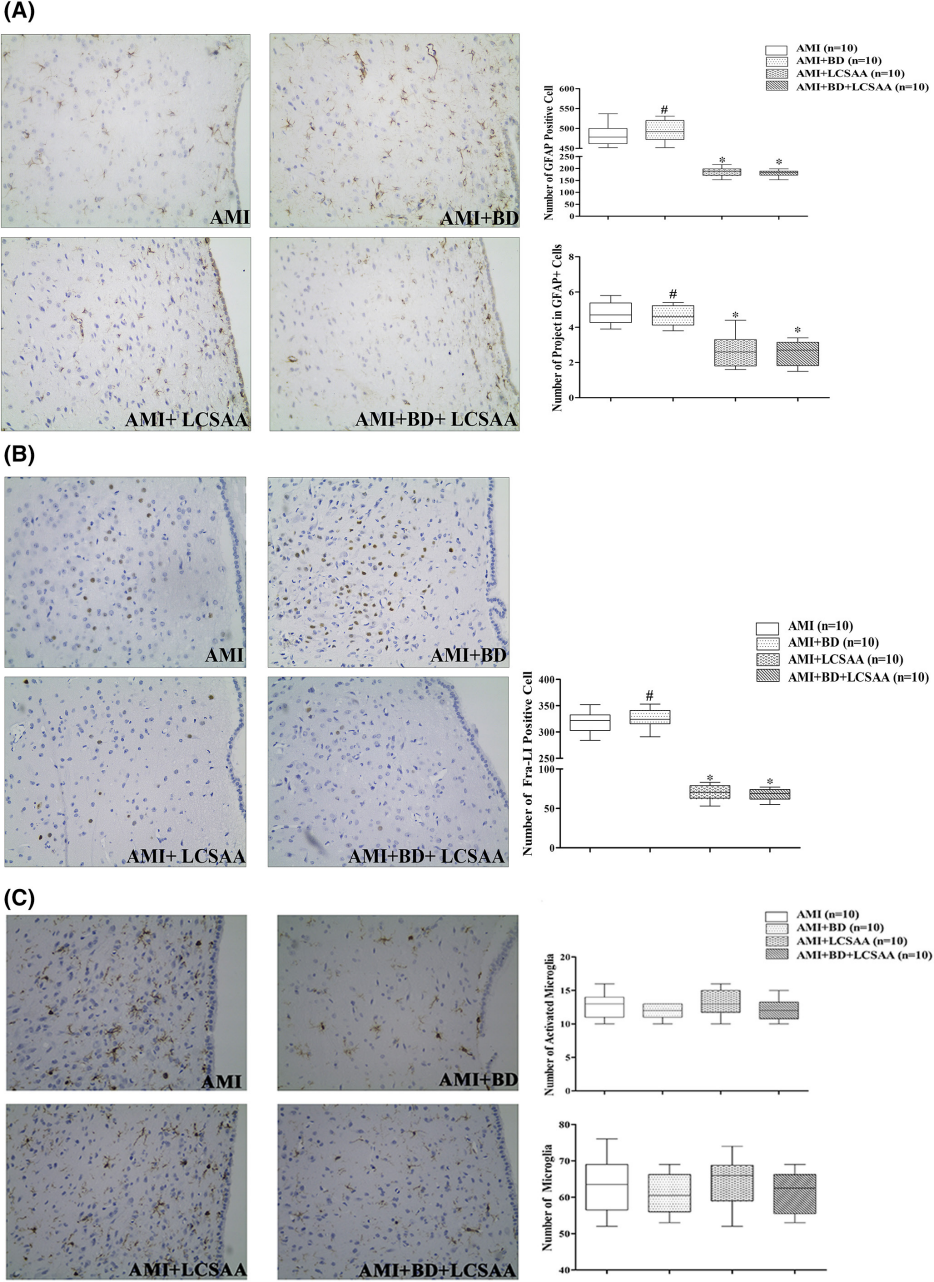
LCSAA attenuated AMI‐induced astrocyte and neuronal activation in the hypothalamus PVN, but had no effect on microglia. (A–C). Representative examples of GFAP‐immunoreactive astrocytes (400×), Fra‐LI positive cells (400×), Iba1 immunohistochemical staining and statistical comparison in bilateral regions of PVN. *P < 0.05 versus AMI group; ^#^ *P* < 0.05 versus the AMI + LCSAA group. AMI, acute myocardial infarction; BD, baroreceptor denervation; LCSAA, local cardiac sympathetic afferent ablation; GFAP, glial fibrillary acidic protein; PVN, paraventricular nucleus; Fra‐LI, Fra‐like immunoreactivity; Iba1, Ionized calcium bindingadaptor molecule‐1

In comparison with the AMI group, less GFAP positive (GFAP+) cells in the PVN exhibited a typical “activated” morphology characterized by cytoplasm hypertrophy with highly ramified projections in AMI + LCSAA or AMI + BD + LCSAA groups (Figure 2A). Morphological quantitative analysis also showed that LCSAA significantly decreases in the number of GFAP+ cells and projections from GFAP+ cells in AMI + LCSAA or AMI + BD + LCSAA rats (*P* < 0.05) (Figure 2A).

As shown in Figure 2B, immunohistochemical study showed that there were fewer Fra‐LI positive cells in the PVN of AMI + LCSAA or AMI + BD + LCSAA rats when compared with AMI rats (*P* < 0.05), but there was no significant difference between AMI + LCSAA group and AMI + BD+ LCSAA group (*P* > 0.05).

### 3.3 Activation of microglia in the PVN

A smaller portion of Iba1 positive cells in four groups exhibited a “amoebic activation” morphology characterized by clearly enlarged soma with considerably thicker and shorter projections (Figure 2C). Moreover, morphological analysis demonstrated that the number of activated microglia or microglia counted in the PVN was not significantly different between four groups (*P* > 0.05) (Figure 2C).

### 3.4 Proinflammatory cytokine in the PVN

**FIGURE 3:**
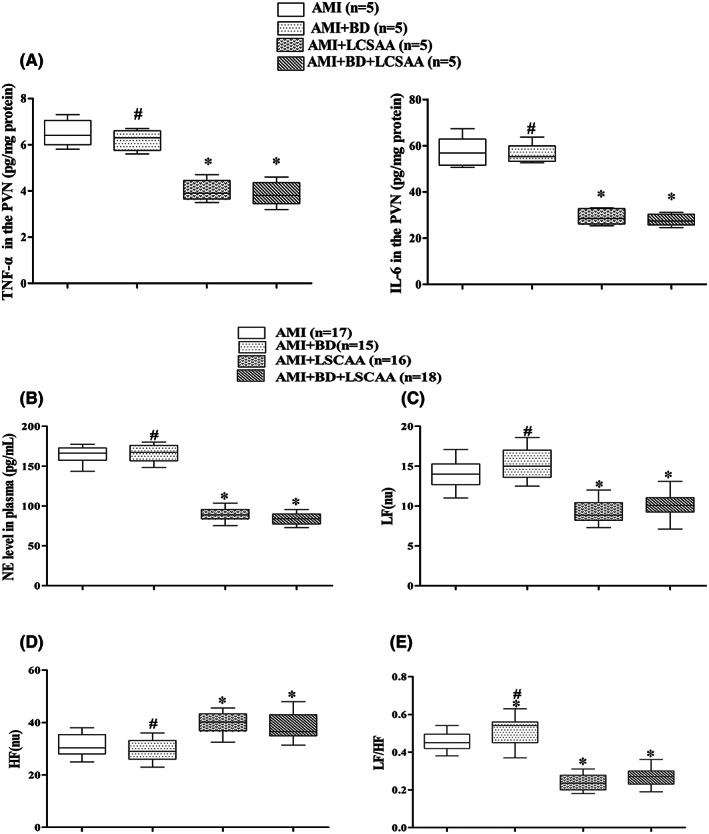
Effect of LCSAA on proinflammatory cytokine in the PVN and sympathetic nerve activity. (A). expression level of TNF‐α and IL‐6 in the PVN. (B). plasma norepinephrine. (C‐E). heart rate variability, including LF, HF and LF/HF. *P < 0.05 versus AMI group; #*P* < 0.05 versus the AMI + LCSAA group. AMI, acute myocardial infarction; BD, baroreceptor denervation; LCSAA, local cardiac sympathetic afferent ablation; NE, norepinephrine; LF, low frequency; HF, high frequency; LF/HF, ratio between LF and HF

TNF‐α and IL‐6 were obviously reduced in AMI + LCSAA and AMI + BD+ LCSAA rats compared with AMI rats (*P* < 0.05). However, there was no significant difference between AMI + LCSAA group and AMI + BD+ LCSAA group or between AMI group and AMI + BD group (*P* > 0.05) (Figure 3A).

### 3.5 Sympathetic nerve activity

Compared with AMI group, plasma NE and the sympathetic components (LF and LF/HF) in HRV were significantly lower and parasympathetic component (HF) was markedly increased following LCSAA but not BD (*P* < 0.05) (Figure 3B–E).

### 3.6 Infarct size, heart rate and blood pressure

**FIGURE 4:**
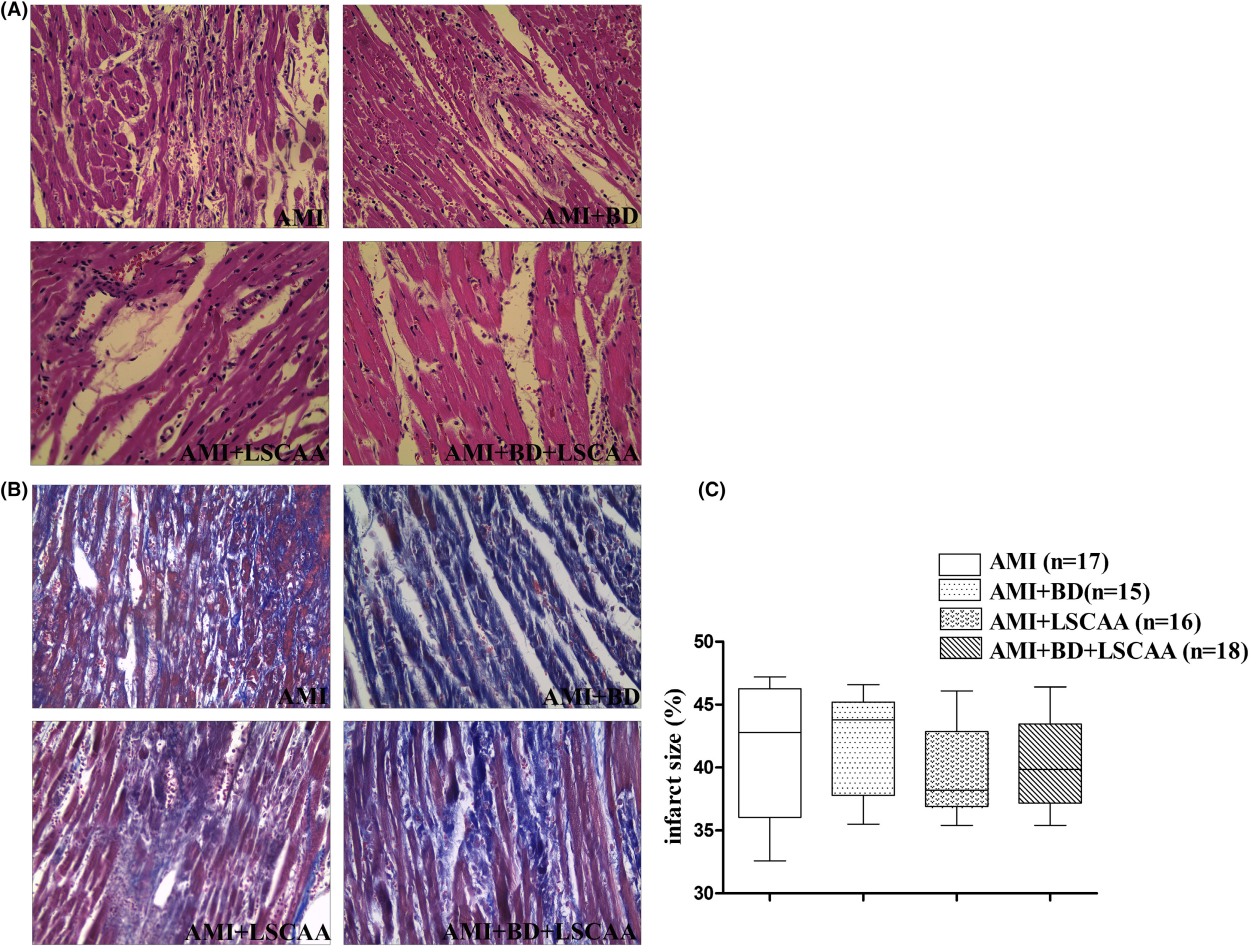
Measurement of infarct size among the four different groups. Representative images of HE stain (A) and Masson's trichrome staining (B) (×400, Scale bar = 40 μm), and infarct size (%) (C). The area stained blue represents infarct size

As shown in Figure 4 and Table 2, there was no obvious difference in infarct size, heart rate and mean arterial pressure among four AMI group (*P >* 0.05).

**TABLE 2:** The change in MAP and HR among the four different groups at 24 h post‐AMI

Group	MAP (mmHg)	HR (beat/min)
AMI (*n* = 17)	76.8 ± 4.7	399.9 ± 12.3
AMI + BD (*n* = 15)	78.5 ± 4.6	401.3 ± 13.8
AMI+ LCSAA (*n* = 16)	74.9 ± 3.5	393.3 ± 14.9
AMI + BD+ LCSAA (*n* = 18)	77.2 ± 5.2	389.5 ± 10.9

Abbreviations: AMI, acute myocardial infarction; BD, baroreceptor denervation; HR, heart rate; LCSAA, local cardiac sympathetic afferent ablation; MAP, Mean arterial pressure.

## 4 DISCUSSION

**FIGURE 5:**
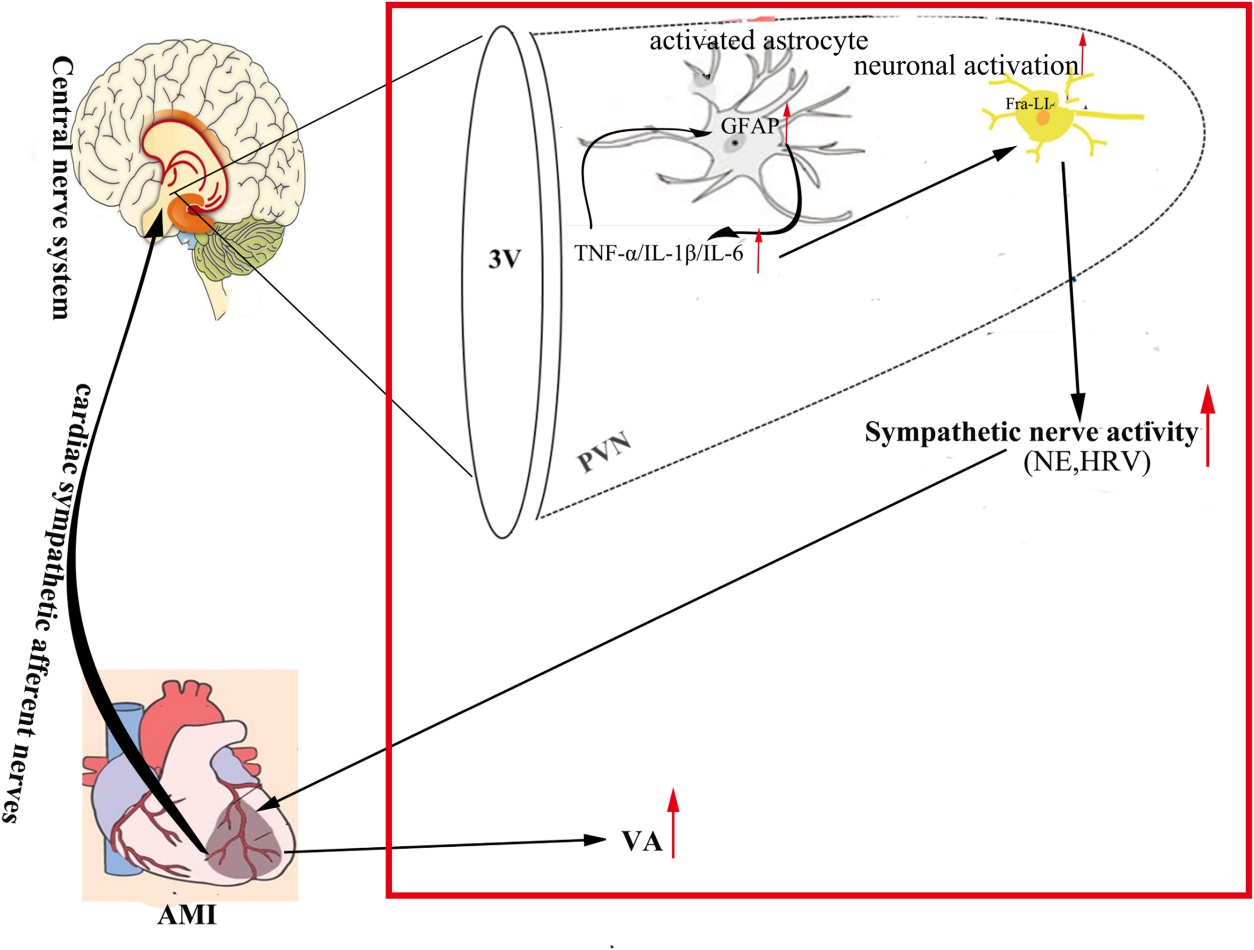
Schematic of the proposed mechanism underlying central regulation of CSAR to prevent VA complicating AMI. Red rectangle indicates the content has been studied previously by our laboratory (Ref. [6]). AMI, acute myocardial infarction; VA, ventricular arrhythmia; PVN, paraventricular nucleus; NE, norepinephrine; HRV, heart rate variability; 3V, third ventricle; GFAP, Glial fibrillary acidic protein; Fra‐LI, Fra‐like immunoreactivity

The present work showed that LCSAA reduced VA occurrence by inhibiting astrocyte and neuronal activation in the PVN at 24 h after AMI, along with deceasing proinflammatory cytokine in the PVN and sympathetic hyperactivation, but BD had no obvious effect. Moreover, activation of microglia in the PVN was not influenced by LCSAA or BD. These results and previous studies indicate astrocyte and neuronal activation in the PVN contribute to CSAR and VA occurrence at 24 h after AMI (Figure 5).

The heart is innervated via the sophisticated neuronal network, especially CSAR. CSAN travel in the superficial epicardium in an apex‐to‐base direction and converges the information from the heart to the brain, while vagal afferent fibres travel deeper in the myocardium until they approach the atrioventricular groove.^12^ Histological and functional research also show that CSAN ending are mainly TRPV1 positive receptors and these afferent nerves are essential for the cardiogenic sympathoexcitatory reflex during myocardial ischemia.^13^ Phenol application to the epicardial surface has been used in selective cardiac sympathetic afferent and efferent denervation.^5^, ^12^ In this study, CSAN was ablated by this method, along with the decreased expression of TRPV1‐expressing afferent nerves. The baroreceptor reflex is also important in regulating cardiovascular activity and may affect the function of CSAR.^4^ In almost all studies, bilateral sinoaortic denervation and cervical vagotomy were usually carried out to eliminate the possible impact of baroreceptor reflex.^11^, ^14^ Thus, we also evaluated the effect of baroreceptor reflex on activated astrocytes in the PVN besides CSAN in this study. As expected, BD had no obvious effect.

CSAR is considered as an important pathway for transmission of cardiac nociception information to brain during myocardial ischemia.^4^ The stimulation of CSAN endings such as bradykinin, adenosine and reactive oxygen species, may induce CASR and thereby increase sympathetic activity.^15^ The enhanced CSAR partially contributes to the sympathoexcitation and pathogenesis of heart failure, hypertension and AMI.^4^, ^16^, ^17^ Zhou et al. reported that cardiac sympathetic afferent denervation could enhance ventricular electrophysiological stability and protect the heart from AMI‐triggered VAs by suppressing left stellate ganglion.^18^ Our previous research suggested that local ablation of the coronary sinus and great cardiac vein (GCV) peripheral nerves could reduce VA in a canine AMI model by decreasing norepinephrine levels in coronary sinus and ventricular tissue.^19^ The central regulation of CSAR may contribute to prevent VA complicating AMI. The previous study has demonstrated that CSAN played an important role in the acute proinflammatory response in the PVN to AMI.^5^ Our recent research showed that activated astrocytes in the PVN increased the proinflammatory cytokines expression at 24 h after AMI.^6^ In this study, we found that the ablation of local CSAN reduced VA occurrence and inhibited the activated astrocytes and proinflammatory cytokine in the PVN. Therefore, astrocytes activation in the PVN may participate in CSAN activity at 24 h after AMI.

Astrocytes are non‐neuronal cells and historically considered as structural supporting cells for neurons, but recently more and more evidence indicated that astrocyte plays an essential role in maintaining proper neuronal health and function.^20^ Astrocytes are closely associated with the pathogenesis of neurodegenerative diseases, mood disorders and energy balance.^21^, ^22^, ^23^ Inflammation‐associated GFAP astrocytes interact with neuronal activation.^8^, ^9^ We also found that neuronal activation and proinflammatory cytokines were reduced following the inhibition of activated astrocytes in the PVN and then VA occurrence was reduced at 24 h after AMI.^6^ In this study, LCSAA could suppress the astrocyte and neuronal activation in the PVN in rats AMI model with no obvious effects on microglia activation, along with deceasing proinflammatory cytokine in the PVN. These results indicate that dynamic astrocyte‐neuron network and proinflammatory cytokine in the PVN may be an underlying central mechanism of CSAR at 24 h after AMI, but detailed mechanisms should be explored in the future.

At present, astrocyte has become a hot research field for neuroscientist and new function are continuously being discovered.^22^, ^24^, ^25^ To our knowledge, the present study is the first to demonstrate that activated astrocytes in the PVN is required for CSAN at the early phase of AMI. Activated astrocytes in the PVN has been reported in association with infarction‐induced heart failure, hypertension and VA complicating AMI by our laboratory and others.^6^, ^26^, ^27^ Our study will provide a new idea for central mechanisms of CSAN, as well as an intact neural pathway for astrocytes activation and sympathoexcitation after AMI.

## 5 LIMITATIONS

There are some limitations in this study. First, the anaesthetics and surgical trauma may affect activated astrocytes, but they were performed in the same manner in all groups and then could be eliminated during analysis. Second, Phenol application to the epicardial surface ablated not only CSAN but also cardiac sympathetic efferent nerve, and the painted region was limited to the distribution of the left anterior descending coronary artery in four group. Lastly, it was very complex in the mechanisms in the brain, for example, how astrocytes in the PVN were activated and how astrocyte‐neuron network interact, these will be explored in future studies.

## 6 CONCLUSIONS

Our study demonstrated that LCSAA could decrease sympathoexcitation and VA occurrence in AMI rats by inhibiting astrocyte‐neuron network and proinflammatory cytokine in the PVN. These findings may provide novel insight into understanding the central regulation of CSAR after AMI.

## AUTHOR CONTRIBUTIONS

**Jugang Chen:** Conceptualization (equal); data curation (equal); formal analysis (equal); methodology (equal); project administration (equal); writing – original draft (lead). **Yingjie Chu:** Formal analysis (equal); writing – original draft (equal). **Meng Gao:** Formal analysis (equal); methodology (equal); project administration (equal). **Xin Dai:** Investigation (equal); project administration (equal). **Bin Li:** Investigation (equal); project administration (equal). **Xiufen Qu:** Conceptualization (lead); supervision (lead); validation (lead); writing – review and editing (lead). **Dechun Yin:** Investigation (equal); project administration (equal); writing – original draft (equal); writing – review and editing (equal).

## CONFLICT OF INTEREST

The authors confirm that there are no conflicts of interest.

## Data Availability

The data that support the findings of this study are available from the corresponding author upon reasonable request.
